# 1134. Lenzilumab Treatment in COVID-19 Pneumonia Reduces Circulating Cytokines and Markers of Systemic Inflammation

**DOI:** 10.1093/ofid/ofac492.973

**Published:** 2022-12-15

**Authors:** Dale Chappell, Adrian Kilcoyne, Frank Cerasoli, John Lukas, Cameron Durrant, Zelalem Temesgen, Christopher Polk, Jason Baker, Vincent Marconi

**Affiliations:** Humanigen Inc., Short Hills, New Jersey; Humanigen Inc., Short Hills, New Jersey; RxMedical Dynamics, New York, New York; CTI, Covington, Kentucky; Humanigen, Inc., Short Hills, New Jersey; Mayo Clinic, Rochester, Minnesota; Atrium Health, Charlotte, North Carolina; Hennepin Healthcare Research Institute, Minneapolis, Minnesota; Emory University, Atlanta, Georgia

## Abstract

**Background:**

Coronavirus disease 2019 (COVID-19) results from SARS-CoV-2-induced hyperinflammatory immune response, orchestrated by granulocyte-macrophage colony-stimulating factor (GM-CSF). GM-CSF increases interleukin-6 (IL-6) levels, ultimately leading to increased C-reactive protein (CRP). The LIVE-AIR trial demonstrated that lenzilumab, the GM-CSF neutralizing antibody, improved the likelihood of survival without invasive mechanical ventilation (IMV) in hospitalized COVID-19 patients requiring supplemental oxygen but not IMV. This sub-analysis correlated levels of cytokines before and after lenzilumab treatment.

**Methods:**

LIVE-AIR was a phase 3, randomized, double-blind, placebo-controlled trial (NCT04351152). Patients hospitalized with COVID-19 pneumonia, requiring only supplemental oxygen, were randomized to receive lenzilumab (1800 mg in three equally divided doses of 600 mg, q8h) or placebo IV infusion, in addition to standard of care which included remdesivir and corticosteroids. Blood taken at baseline (BL) and subsequent to treatment through day 10 (D10) were obtained and analyzed by high sensitivity enzyme immunoassay for GM-CSF, IL-6, and CRP.

**Results:**

Baseline IL-6 levels (Log_e_-transformed for all cytokines and biomarkers) were linearly correlated with higher baseline GM-CSF levels (slope=0.60, p< 0.001). Baseline CRP levels were linearly correlated with higher baseline IL-6 levels (slope=0.29, p < 0.001). GM-CSF levels decreased with lenzilumab treatment on day 1 (D1) which persisted through D10 (Table). In contrast, GM-CSF increased with placebo treatment. IL-6 levels decreased only with lenzilumab treatment. CRP following lenzilumab or placebo treatment decreased on D1 to similar levels and further decreased on D10 only with lenzilumab treatment.

Cytokine Levels Associated with Lenzilumab Treatment

**Figure:**
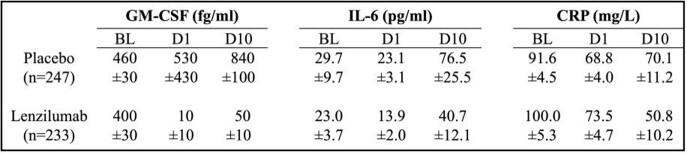

**Conclusion:**

Lenzilumab decreased GM-CSF as well as downstream cytokines and systemic biomarkers of inflammation during the hyperinflammatory immune response of COVD-19.

**Disclosures:**

**Dale Chappell, MD, MBA**, Humanigen, Inc: Employee|Humanigen, Inc: Ownership Interest **Adrian Kilcoyne, MD**, Humanigen, Inc: Employee **Frank Cerasoli, PhD**, Humanigen, Inc: Advisor/Consultant **John Lukas, PhD**, Humanigen, Inc: Advisor/Consultant **Cameron Durrant, MD**, Humanigen, Inc: Employee|Humanigen, Inc: Ownership Interest **Zelalem Temesgen, MD**, Gilead: unrestricted educational grant (to the institution)|Humanigen, Inc: Grant/Research Support|Merck: unrestricted educational grant (to the institution)|ViiV: Advisor/Consultant **Christopher Polk, MD**, Gilead: Advisor/Consultant **Jason Baker, MD**, Gilead: Grant/Research Support|Humanigen, Inc: Grant/Research Support **Vincent Marconi, MD**, Gilead: Grant/Research Support.

